# Neuroprotective effects of morroniside from *Cornus officinalis* sieb. *Et zucc* against Parkinson’s disease via inhibiting oxidative stress and ferroptosis

**DOI:** 10.1186/s12906-023-03967-0

**Published:** 2023-07-01

**Authors:** Mao Li, Junli Zhang, Lianyan Jiang, Wujun Wang, Xianrong Feng, Meijun Liu, Dongdong Yang

**Affiliations:** 1 The First Affiliated Hospital of Henan University of Traditional Chinese Medicine, Zhengzhou, China; 2 Hospital of Chengdu University of Traditional Chinese Medicine, Chengdu, China

**Keywords:** Parkinson’s disease, Morroniside, Mitochondria, Ferroptosis, Oxidative stress

## Abstract

Parkinson’s disease (PD) is the second most common neurodegenera­tive disorder after Alzheimer disease accompanied by the death of dopaminergic neurons and brain nigrostriatal mitochondrial damage in the elderly population. The features of the disease include tremor, rigidity, postural instability, and motor retardation. The pathogenesis of Parkinson’s disease is complex, and abnormal lipid metabolism resulting in ferroptosis due to the excessive accumulation of free radicals from oxidative stress in the substantia nigra of the brain was thought to be one of the factors causing the disease. Morroniside has been reported to have significant neuroprotective effects, although it has not been studied in PD. Therefore, this study focused on determining the neuroprotective effects of morroniside (25, 50, and 100 mg/kg) on 1-methyl-4-phenyl-1,2,3,6-tetrahydropyridine (MPTP, 30 mg/kg)-induced mice models of PD and explored 1-methyl-4-phenylpyridinium MPP+-induced ferroptosis in PC12 cells. Morroniside restored impaired motor function in the PD mice models while reducing neuronal injury. The activation of nuclear factor erythroid 2-related factor 2/antioxidant response elements (Nrf2/ARE) by morroniside promoted antioxidation, the content of reducing agent glutathione (GSH) increased, and the level of the lipid metabolite malondialdehyde (MDA) decreased. Notably, morroniside inhibited ferroptosis in substantia nigra of the brain and PC12 cells, reduced iron levels, and upregulated the expression of the iron-regulated proteins glutathione peroxidase 4 (GPX4), solute carrier family 7 member 11 (SLC7A11), ferritin heavy chain 1 (FTH-1), and ferroportin (FPN). More importantly, morroniside repaired the mitochondrial damage, restored the mitochondrial respiratory chain, and inhibited the production of reactive oxygen species (ROS). These data indicated that morroniside could activate the Nrf2/ARE signaling pathway to increase the antioxidant capacity, thereby inhibiting abnormal lipid metabolism and protecting dopaminergic neurons from ferroptosis in PD.

**Supplementary Information:**

The online version contains supplementary material available at 10.1186/s12906-023-03967-0.

## Introduction

Parkinson’s disease (PD) is the second most common neurodegenerative disorder after Alzheimer disease [1–4], characterized by degenerative necrosis of dopaminergic neurons in the brain, which causes a decrease in the relative levels of dopaminergic neurotransmitters in the brain and induces a series of extrapyramidal symptoms such as bradykinesia, rigidity, and tremor [5, 6]. Mitochondrial damage is critical in the pathogenesis of degenerative diseases of the central nervous system and has emerged as a new research hotspot and potential target for treatment [7]. With the increasing age of the world’s population, the incidence of PD is increasing. Dopamine replacement therapy is the main method but has toxic side effects and serious drug resistance [8, 9]. PD has become a challenge in the global medical field, and there is an urgent need to develop novel drugs and treatments.

The pathogenesis of Parkinson’s disease is complex and involves a combination of factors. Studies have reported that the excessive accumulation of free radicals produced by oxidative stress can damage the nigrostriatal dopaminergic neurons in the brain, resulting in reduced secretion of dopaminergic neurotransmitters and producing a range of symptoms of motor impairment [10]. Nuclear factor erythroid 2-related factor 2 (Nrf2) is a major transcription factor that is highly sensitive to redox environment and binds to the antioxidant response elements (ARE), thereby initiating downstream antioxidant enzymes [11]. ARE upregulates the proteins of NAD(P)H quinone oxidoreductase 1 **(**NQO1) and heme oxygenase 1 (HO-1) in the brain substantia nigra of PD patients, indicating the activation of the Nrf2-ARE signaling pathway [12, 13]. However, Nrf2-activated gene transcription can be suppressed by multiple factors. When the PD-associated DJ-1 protein is deficient, the Nrf2 protein is unstable and transcriptional responses are highly susceptible to blockage by multiple factors such as reactive oxygen species (ROS) [14]. The reduced antioxidant capacity of the body and the accumulation of lipid-ROS can cause ferroptosis [15]. Bruce reported that ferroptosis in the PD mouse model prepared by 1-methyl-4-phenyl-1,2,3,6-tetrahydropyridine (MPTP), associated with the activation of protein kinase Cα **(**PKCα) and ferrostatin-1 (ferroptosis inhibitor), significantly inhibited the toxicity of MPTP on dopaminergic neurons [16]. Thus, drugs capable of modulating the level of oxidative stress in the body and avoiding cellular ferroptosis may potentially alleviate PD.

Ferroptosis is an important pathological process in the degenerative death of dopamine neurons and is closely linked to various pathological mechanisms such as oxidative stress and mitochondrial dysfunction as the study progressed [17]. Under normal physiological conditions, iron is transported between the cytoplasm and the mitochondria, and mitochondrial dysfunction disrupts iron metabolism, eventually leading to ferroptosis [18]. Ferroptosis is an iron- and lipid ROS-dependent form of programmed cell death [19]. Several factors contribute to the pathogenesis of PD, including mitochondrial dysfunction and the production of large amounts of superoxide radicals, which disrupt mitochondrial iron metabolism and eventually leads to ferroptosis in dopamine neurons [20].

Morroniside belongs to the cyclic enol ether glycosides and is the active ingredient in the traditional Chinese medicine *Cornus officinalis* Sieb. *Et Zucc* [21]. Morroniside has been reported to exert neuroprotective, antioxidant, and anti-inflammatory effects [22–24]. Studies have reported that morroniside can enhance the activity of superoxide dismutase (SOD) and glutathione in ischemic cortical tissue, decrease the expression of caspase-3, and protect against focal cerebral ischemic injury in rats [25]. Recent studies on morroniside have focused on its neuroprotective effects, among which, its use in the treatment of cerebral ischemia-reperfusion injury is more common. However, none of the studies have looked at the effect of morroniside on PD.

In this paper, we subjected PD mice models to open-field and pole-climbing experiments, analyzed the organization and substructure of their brain substantia nigra, and analyzed the level of tyrosine hydroxylase (a key enzyme for dopamine synthesis, TH). The level of oxidative stress and ferroptosis in the substantia nigra of mice were studied. The effect of morroniside on the oxidative stress pathway Nrf2/ARE and ferroptosis was verified in PC12 cells. The protective effect of morroniside against mitochondrial damage was investigated. To our knowledge, this study represents the first report to date on the association of morroniside with PD and provides novel information on the modulation of PD by morroniside.

## Materials and methods

### Animals and grouping

Thirty-six healthy male C57BL/6 mice of SPF grade aged 8–12 weeks and weighing 22–32 g were purchased from the Chengdu Dashuo Experimental Animal Co. Ltd. (certificate SCXK(Chuang) 2020-030, Chengdu, Sichuan, China). The mice were housed at 23 ± 2 °C, fed ad libitum, exposed to natural light, and were acclimatized for one week. The mice were divided into 6 groups (n = 6): control group, model group, morroniside low (L-morroniside), medium (M-morroniside), and high (H-morroniside) dose group, and positive drug madopar group. The control group was injected with the same dose of saline as the MPTP-induced PD model group. The PD model was induced by intraperitoneal injection of MPTP (1-methyl-4-phenyl-1,2,3,6-tetrahydropyridine, AbMole, USA) at 30 mg/kg for 5 consecutive days. Based on the model group, the mice were divided into morroniside (HPLC ≥ 97%, Aktin Chemicals, Inc. China) low, medium, high groups, and madopar (Guiechem, China) group. At the beginning of modeling, the mice were given 25, 50, and 100 mg/kg morroniside via gavage, along with 50 mg/kg·d madopar (Guiechem, China) to the madopar group for 14 d. After the completion of the experiments, the animals were given a 50 mg/kg intraperitoneal injection of 1% sodium pentobarbital. To obtain the substantia nigra tissues, the animals were sacrificed. All experimental procedures were carried out under the guidelines of the Animal Experimental Committee and the Ethics Committee of Chengdu University of Traditional Chinese Medicine (No. 20,211,498 A).

### Open field experiment

The behavioral differences of experimental mice were studied according to the classical method of open field studies [26]. The open field consisted of 70 × 70 × 30 cm melamine boxes (Shanghai XinRuan Information Technology Co. Ltd, China). The box was divided into 16 equal squares and two areas (middle and outer perimeter). Each mouse was placed in the central area and continuously monitored for 5 min to analyze the time and frequency of each mouse entering different areas, and the mouse feces were disposed of promptly during the experiment to reduce the disturbance of odor.

### Pole test

The lower end of the log climbing rod was fixed, with a diameter of 0.8 cm and a length of about 40 cm. The mice were placed on the top of the climbing bar (Shanghai XinRuan Information Technology Co. Ltd, China) and guided to climb down from top to bottom within 15 s. After 1 h of administration of the drug by gavage, the pole-climbing time of mice was measured. The mice were placed head down on the top of the pole and allowed to climb down naturally. The time taken by the mice from the top of the pole until both forelimbs touched the bottom platform of the pole was recorded and repeated thrice [27].

### HE staining

Hematoxylin-eosin (HE, Solarbio, China) staining was performed for substantia nigra studies. The substantia nigra of the brain was fixed in 4% paraformaldehyde, dehydrated, and embedded in paraffin. Subsequently, the substantia nigra was cut into 4 μm thick sections, dewaxed, and rehydrated. The slides were immersed in hematoxylin for 2 min and then rinsed in water for 1 min. Then, the slides were stained with 1% eosin solution and incubated for 3s. The slices were dehydrated twice in 95% ethanol and 100% ethanol for 3 min. After a final treatment with xylene and sealing with resin, the photos were taken under an optical microscope (MOTIC, Hong Kong, China).

### Transmission electron microscopy assay

The substantia nigra was removed from each group of mice, fixed in 5% glutaraldehyde for 5 h at 4 °C, and washed thrice with neutral phosphate buffer for 10 min each time. Then, the tissue was fixed in 0.1 mol/L osmium acid for 3 h and washed again in phosphate buffer thrice for 10 min each time. Then, the gradient dehydration was performed according to 50%, 70%, 80%, 90%, 95%, and 100% ethanol for 15 min each time. The resin was impregnated, embedded, polymerized, and then made into 60–80 nm ultra-thin sections, double-stained with uranium-lead, dried at room temperature, and photographed under a transmission electron microscope (TEM, JEOL, Japan).

### Immunohistochemistry assay

Substantia nigra Sect. (5 μm) from each group were dewaxed and dehydrated. Then, the sections were treated with 3% BSA for 1 h at 25 °C and incubated with rabbit anti-mouse tyrosine hydroxylase (TH) (1:100, ab75875, Abcam, UK) overnight at 4 °C. After washing the sections with PBS, they were incubated with goat anti-rabbit IgG antibody (1:500, s0001, Affinity, AUS) at room temperature for 2 h, stained with diaminobenzidine (DAB, Solarbio, China), and counter-stained with hematoxylin. The TH-positive area of the substantia nigra was observed under a microscope, and five fields were randomly selected for image analysis. Immunohistochemical images were quantified using Image J (NIH, USA) analysis software. Mean density, which represents the expression of protein, is the cumulative integrated optical density (IOD) divided by the area of the effective target distribution.

### Prussian blue reaction assay

Paraffin sections of substantia nigra were dewaxed in water, and the sections were sequentially placed in xylene I for 20 min, xylene II for 20 min, anhydrous ethanol I for 5 min, anhydrous ethanol II for 5 min, 75% ethanol for 5 min, washed with water, and then with distilled water thrice. The potassium ferrous cyanide solution and hydrochloric acid solution were then mixed in equal proportions into a Prussian blue dye solution (Solarbio, China), and the tissues were stained for 1 h before being washed twice with distilled water. The tissue was then stained for 5 min in a nuclear fast red solution (0.1%, Solarbio, China) before rinsing with running water. Finally, the slides were placed in anhydrous ethanol I for 5 min, anhydrous ethanol II for 5 min, anhydrous ethanol III for 5 min, xylene I for 5 min, xylene II for 5 min, and neutral resin for 5 min to seal the slides. The stained slides were then subjected to microscopic examination, image acquisition, and analysis.

### Immunofluorescence assay

Paraffin sections of substantia nigra were dewaxed in water and incubated with 3% BSA and 0.3% Triton for 2 h at room temperature to block the antigen. After washing with PBS, the sections were incubated with rabbit anti-mouse Nrf2 antibody (1:400, Abcam, UK) at 4 °C for 24 h and Alexa Fluor488 treatment-labeled goat anti-rabbit IgG (1:400, Abcam, UK) at room temperature for 2 h in the dark. After washing with PBST, the sections were imaged with a laser confocal fluorescence microscope (Leica, Germany). To quantify the immunofluorescence intensity, the integrated optical density (IOD) was calculated using Image J software, and the mean IOD (MOD) was calculated from IOD/Area.

### Detection of intracellular ferrous iron

Intracellular ferrous iron level in the substantia nigra or PC12 cells was checked with an iron assay kit (ml095089, Shanghai Enzyme-linked Biotechnology Co. Ltd., China) according to the manufacturer’s instructions. The tissue or cells were washed twice with cold PBS, and lysed in 200 µL lysis solution without EDTA, citrate, or other metal chelating agents and placed on a shaker for 2 h. Then, 200 µL of the sample was added into a 96-well plate, and the absorbance was measured. Ferrous iron reacts with Ferene S to produce a stable-colored complex that can be accessed immediately by reading the absorbance at 593 nm [28].

### Elisa

The mouse substantia nigra or treated PC12 cells were collected and lysed with an appropriate amount of lysis solution. The supernatant was extracted by centrifugation at 3000 rpm for 10 min at 4 °C. A dilution of 0.1 mL of the sample was added to the reaction wells and incubated at 37 °C for 1 h. The specific operation and content calculation were then performed according to the manufacturer’s instructions (Jining Shiye, China).

### Western blot

The mouse substantia nigra or PC12 cells were lysed on ice in RIPA buffer (Beyotime, China). The lysates were centrifuged, the supernatant was collected, and the total protein concentration was determined by BCA (P0013, Beyotime, China). The protein samples were electrophoresed on 8–14% SDS gels and transferred to the PVDF membrane. Subsequently, the membranes were incubated in TBST (Tween-20) containing 5% skim milk at 25 °C. The membranes were rinsed with TBST and then incubated overnight with primary antibodies Nrf2 (1:500, A0674, abclonal, China), HO-1 (1:500, A19062, abclonal, China), GPX4 (1:1000, ab125066, abcam, UK), SLC7A11 (1:1000, ab175186, abcam, UK), FTH-1 (1:1000, ab183781, abcam, UK), FPN (1:500, A14884, abclonal, China) using β-actin (1:500, AC026, abclonal, China) as the internal reference protein. Thereafter, the membranes were rinsed with TBST and incubated with a secondary antibody for 1 h. After washing, the protein expression levels were detected by an enhanced chemiluminescence detection kit (KF001, Affinity, AUS).

### Quantitative real-time polymerase chain reaction (qRT-PCR)

The TRIzol RNA extraction kit (Invitrogen, USA) was used to collect total RNA from PC12 cells and nigra tissue. Gel electrophoresis and NanoDrop 2000 spectrophotometry (Thermo, USA) were used to determine the RNA’s integrity and purity. The reverse transcription was carried out using a reverse transcription kit (Invitrogen, USA) according to the manufacturer’s instructions. qRT-PCR was used to quantify the resulting cDNA, which was then evaluated using a Qubit fluorescence spectrometer (Invitrogen, USA). The primer sequences are presented in Table 1.

**Table 1:** Sequences of primers used in qRT-PCR

Target Gene		Primers (5′ to 3′)
GPX4	forward	CTCCATGCACGAATTCTCAG
	reverse	ACGTCAGTTTTGCCTCATTG
SLC7A11	forward	CCTGGCATTTGGACGCTACA
	reverse	GCAAGGGGGATGGTTTTTTC
GADPH	forward	CGTGTTCCTACCCCCAATGT
	reverse	TGTCATCATACTTGGCAGGTTTCT
GPX4	forward	AATTCGCAGCCAAGGACATCG
	reverse	ATTCGTAAACCACACTCGGCGTA
SLC7A11	forward	TGCTGCCTACACAAAGACGTT
	reverse	CGCCTTGCCCTTTAAGTATTCACC
β-actin	forward	ACATCCGTAAAGACCTCTATGCC
	reverse	TACTCCTGCTTGCTGATCCAC

### Cell culture

The PCI2 cells were obtained from the Chinese Academy of Medical Sciences. They were cultured in a DMEM (Gibco, USA) medium containing 10% fetal bovine serum (Gibco, USA), 100 mg/L streptomycin, and 100,000 U/L penicillin (MCE, USA) at 37 °C in a 5% CO_2_ incubator. When the degree of fusion of PC12 cells reached 80% or more, they were digested using 0.25% trypsin for about 2 min, and a serum-containing medium was used to terminate the digestion. The cells were then centrifuged at 1000 rpm for 5 min and passaged in a serum-containing fresh medium.

### CCK-8 assay

The PC12 cells at the logarithmic growth stage were inoculated with 5 × 10^4^ cells/mL and incubated in 96-well plates for 24 h at 37 °C. Then, 1 mmol/L MPP^+^ (1-methyl-4-phenyl pyridine, D048, sigma, US) was used to induce ferroptosis in the PC12 cells. After treatment with morroniside or 2µM ML385 (B83002122EF46, APEXBIO, US) for 24 or 48 h, 10 µL CCK-8 was added to each well and incubated for 3 h. The absorbance values of each well were measured at 570 nm on an enzyme marker (Thermo Scientific, USA).

### Flow cytometry for ROS

The PC12 cells were divided into control, morroniside, and MPP^+^-induction groups. The Nrf2 inhibitor ML385 or morroniside was used in combination with MPP^+^. About 1 × 10^7^ cells were taken from each group and washed twice with PBS. Before probe loading, DCFH-DA (S0033S, beyotime, China) was diluted with serum-free culture solution at 1: 1000 to a final concentration of 10 µM and immersed for 30 min at 37 °C in the dark. Further, the cells were centrifuged and washed 1 to 2 times with a serum-free cell culture medium to remove the DCFH-DA that had not entered the cells adequately. The cells were loaded with probes, prepared as 500 µL suspensions to make a cell density of 1 × 10^6^ to 2 × 10^7^. The intensity of the fluorescence of PC12 cells was measured in real-time using an excitation wavelength of 488 nm and an emission wavelength of 525 nm. The FITC channel was used to monitor the changes in levels of ROS in different groups.

### Statistical analysis

All values were expressed as means ± SD, and the analysis was performed using SPSS version 23.0 software. One-way ANOVA was used to determine the differences in mean values. If the data fit the homogeneity of variance, LSD analysis was selected. Otherwise, Tamhane’s T2 analysis was selected. *P* < 0.05 was considered statistically significant.

## Results

### The effect of morroniside on the behavior of MPTP-induced PD mice

**Fig. 1:**
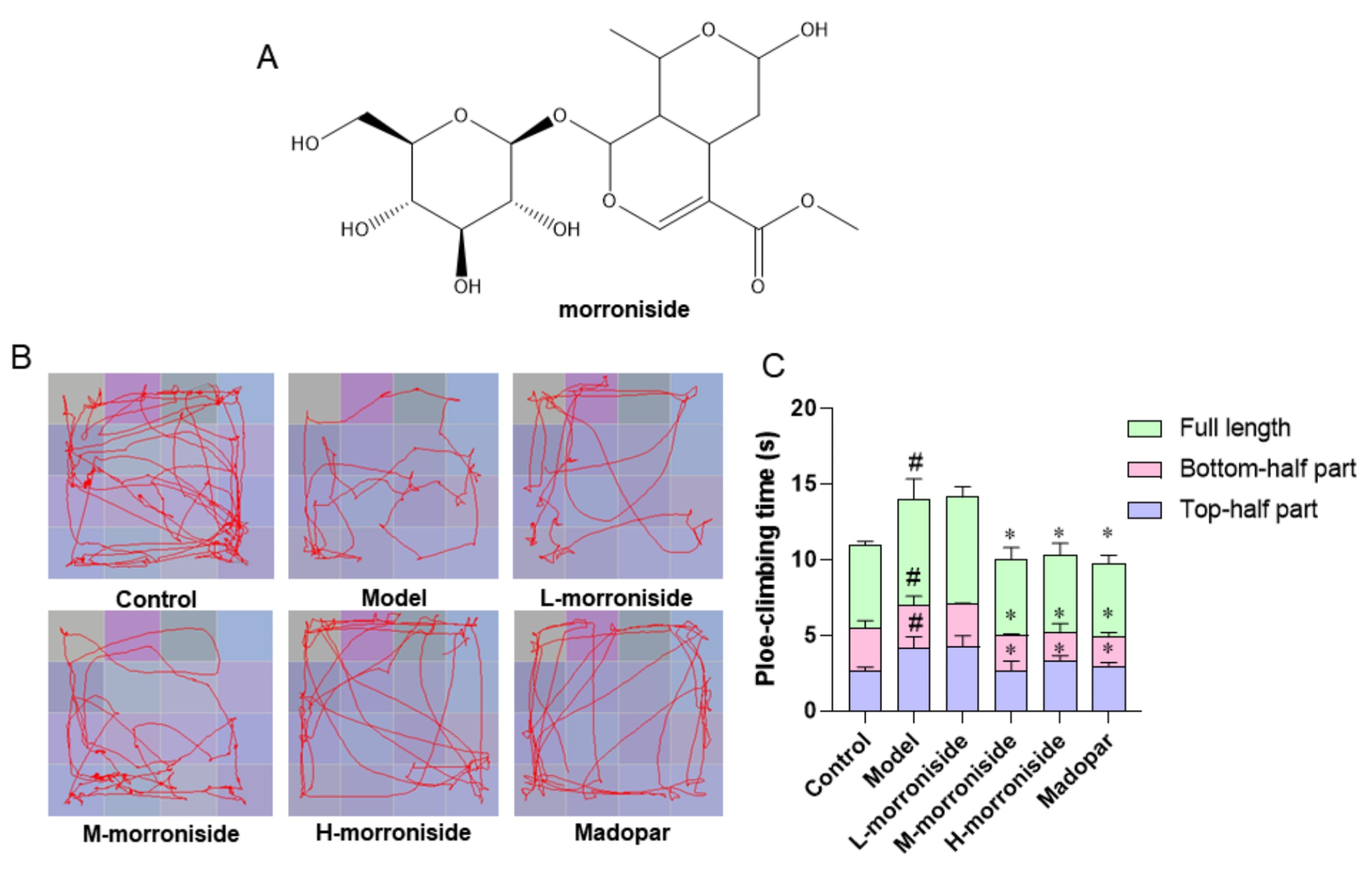
Morroniside improves the behavior of PD mice. **(A)** Chemical structure of morroniside. **(B)** Open-field test. **(C)** Pole-climbing test. Data are shown as mean values ± SD, n = 6 per group, ^#^*p* < 0.05 compared to the control group. *p < 0.05 compared to the model group

C57BL/6 mice showed reduced locomotion, limb stiffness, unsteady gait, and delayed response after MPTP administration, which matched the clinical manifestations of PD patients. Morroniside is a natural product with the chemical structure shown in Fig. 1A. The effect of morroniside on the behavioral profile of PD mice was examined by the Open-field test and the Pole-climbing test. The open-field experiments revealed that the model group mice preferred the peripheral region more than the control group. Compared with the model group, the number of uprights, inter-square crossings, central grid crossings, and central grid dwell time increased with increasing concentration of morroniside in the morroniside group. Furthermore, the madopar group behaved similarly to the high-dose morroniside group (Fig. 1B). The pole-climbing experiment revealed that the mice in the model group took longer to climb the pole than the mice in the control group. Compared to the model group, low doses of morroniside had no significant effect on the behavior of PD mice, while medium and high doses of morroniside and madopar significantly reduced the pole-climbing time (*P* < 0.05) (Fig. 1C). These results suggested that morroniside significantly improved the behavior of MPTP-induced PD mice.

### Effect of morroniside on histopathological changes and TH levels in the brain substantia nigra of PD mice

**Fig. 2:**
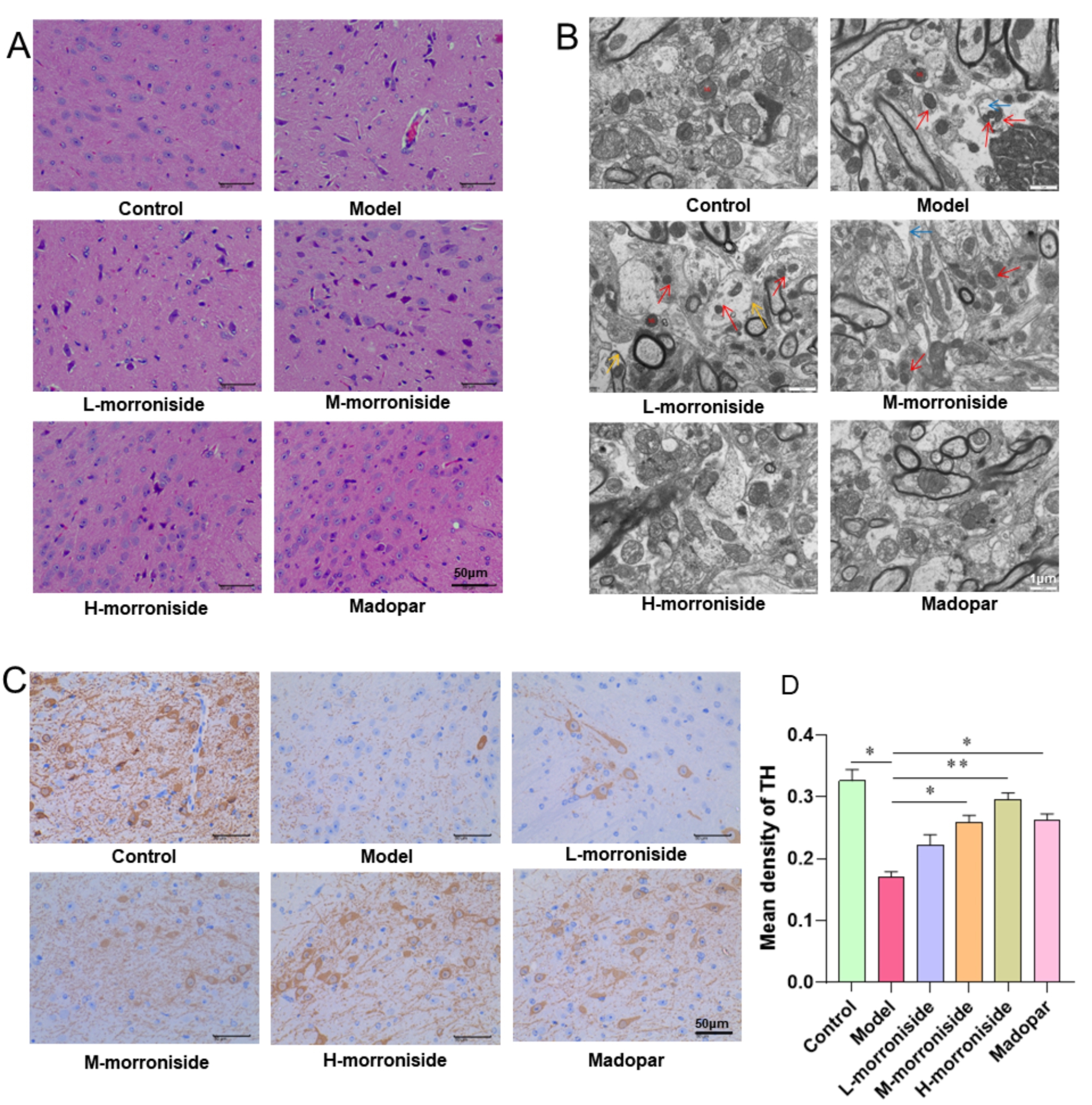
Morroniside improves the structural damage and TH levels in the substantia nigra of PD mice. **(A)** HE staining of the substantia nigra (n = 3 per group). **(B)** Ultrastructure of the substantia nigra under TEM (red arrows indicate mitochondrial fixation and blue arrows indicate nerve fiber lysis, n = 3 per group). **(C)** IHC for TH in the substantia nigra (n = 6 per group). **(D)** TH expression level statistics (n = 6 per group). Data are shown as mean values ± SD, *p < 0.05, **p < 0.01

The most important pathological change in PD is the degeneration and necrosis of dopaminergic neurons in the substantia nigra of the brain. The results of the HE staining indicated that compared to the control group, neurons in the model group were reduced, the nucleus was pyknotic and irregular, the darker staining indicated degeneration and necrosis, and the number of surrounding microglia increased. The morroniside group could alleviate this lesion in a dose-dependent manner compared to the model group. In the high-dose morroniside and madopar groups, the neurons in the substantia nigra had normal morphology, large nuclei, and clear outlines, while the structure of the glial cells was clearer, with no neuronal phagocytosis or satellite phenomena (Fig. 2A). The TEM results revealed that compared to the control group, few nerve fibers in the axons of the model group were slightly dissolved, most mitochondria were contracted, the gaps between the crests widened, the membrane density increased, and neuronal cell apoptosis occurred. Most of the mitochondria in the low-and middle-dose morroniside groups had pyknosis compared to the model group. Moreover, in the high-dose morroniside and madopar groups, the axons were rich in nerve fibers, and the mitochondrial morphology was relatively normal (Fig. 2B). The immunohistochemistry results indicated that the expression of TH in the model group was significantly lower when compared to the control group (*P* < 0.05). Morroniside, when compared to the model group, can induce TH expression in a dose-dependent manner. Furthermore, at high doses of morroniside and madopar, the level of TH was comparable to that of the control group (Fig. 2C and D). Morroniside was found to improve tissue and organelle damage repair in PD mice and promote the recovery of TH levels in the brain’s substantia nigra.

### Effect of morroniside on ferroptosis in the substantia nigra of PD mice

**Fig. 3:**
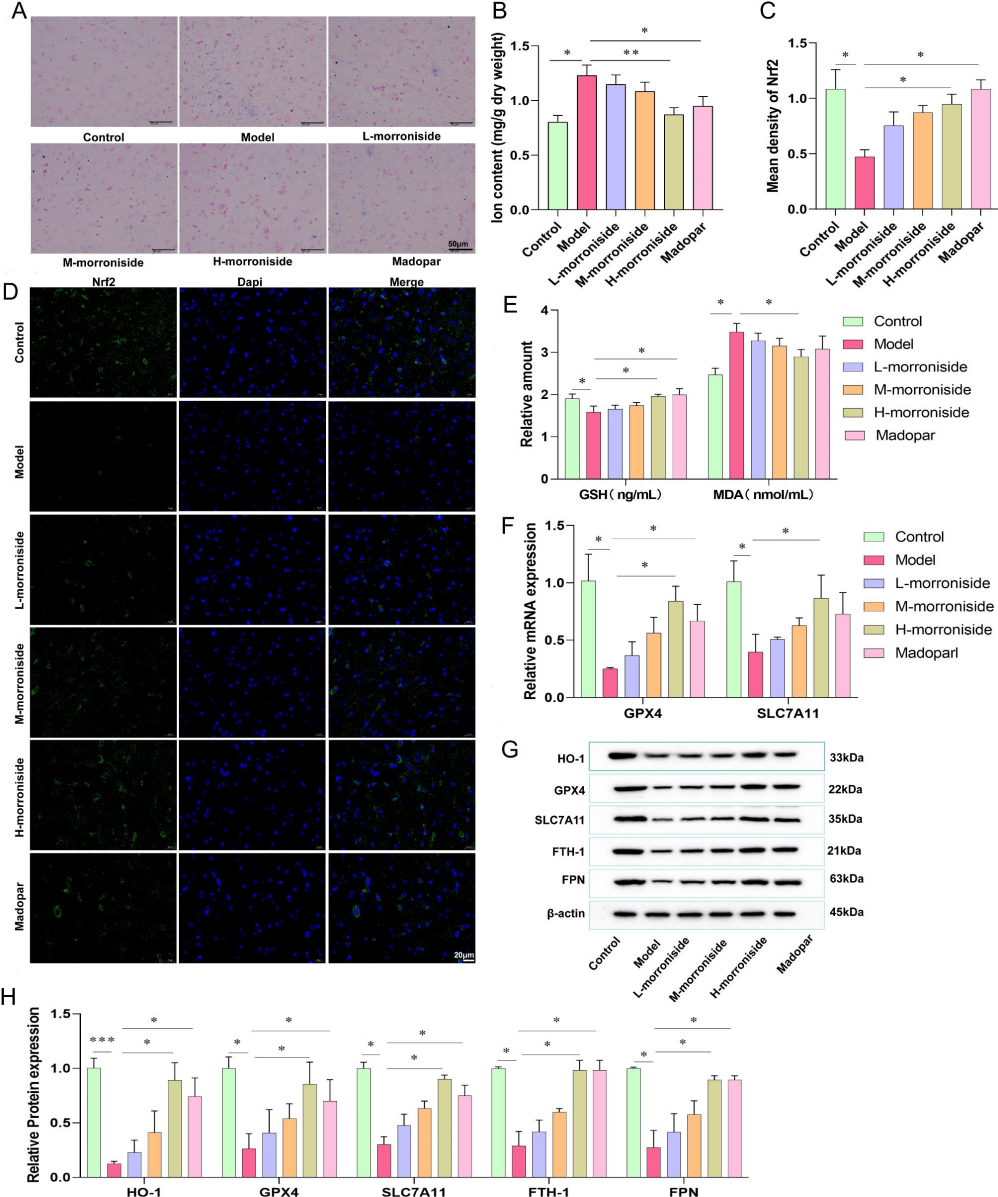
Morroniside regulates ferroptosis in the substantia nigra cells. **(A)** Prussian blue iron staining of the substantia nigra. **(B)** Ferrous iron in substantia nigra. **(C)** Relative expression of Nrf2. **(D)** Immunofluorescence for the detection of Nrf2. **(E)** Levels of GSH and MDA in substantia nigra. **(F)** Relative mRNA expression of GPX4 and SLC7A11 using qRT-PCR. **(G)** HO-1, GPX4, SLC7A11, FTH-1, and FPN in substantia nigra by Western blot. **(H)** Relative expression of HO-1, GPX4, SLC7A11, FTH-1, and FPN. Data are shown as mean values ± SD, n = 6 per group, *p < 0.05, **p < 0.01, ***p < 0.001

The reduced antioxidant capacity of cells and the accumulation of lipid-reactive oxygen species caused ferroptosis. Nrf2 is important in the pathogenesis of PD, which can regulate the antioxidant response of the body. The results of the Prussian blue staining indicated that the percentage of positive-expression area in the model group significantly increased compared to the control group. Compared with the model group, the percentage of positive-expression area in the low-dose group was also decreased, although it was not statistically significant (*P* > 0.05). The percentage of positive-expression area in the middle-dose, high-dose, and madopar groups decreased by varying degrees, which was statistically significant (*P* < 0.05) (Fig. 3A). Similarly, the ferrous iron content in the substantia nigra of the brain was significantly higher in the model group than in the control group. Morroniside could reduce ferrous iron content levels in the substantia nigra in a dose-dependent manner. Furthermore, the effects of high doses of morroniside and madopar were similar to those observed in the control group (Fig. 3B). The transcription factor Nrf2 in the substantia nigra of the brain was significantly lower in the model group compared to the control group. However, morroniside increased Nrf2 expression and had a dose-dependent effect, whereas madopar had a similar effect as high doses of morroniside (Fig. 3 C and D). The GSH decreased in the model group compared to the control group (Fig. 3E). As shown in Fig. 3F, morroniside and madopar restored aberrant GPX4 and SLC7A11 mRNA levels. The GSH was significantly higher in the high-dose morroniside and madopar groups compared to the model group. Oxidative stress and ferroptosis-related proteins are presented in Fig. 3G and H. Compared with the control group, the expressions of HO-1, GPX4, SLC7A11, FTH-1, and FPN were significantly downregulated in the model group. Morroniside can upregulate the MPTP-induced decrease of HO-1, GPX4, SLC7A11, FTH-1, and FPN and madopar has similar effects as morroniside. These results suggested that morroniside could affect the expression of GPX4, SLC7A11, FTH-1, and FPN mediated ferroptosis.

### Morroniside regulates levels of oxidative stress in PC12 cells via Nrf2-ARE

**Fig. 4:**
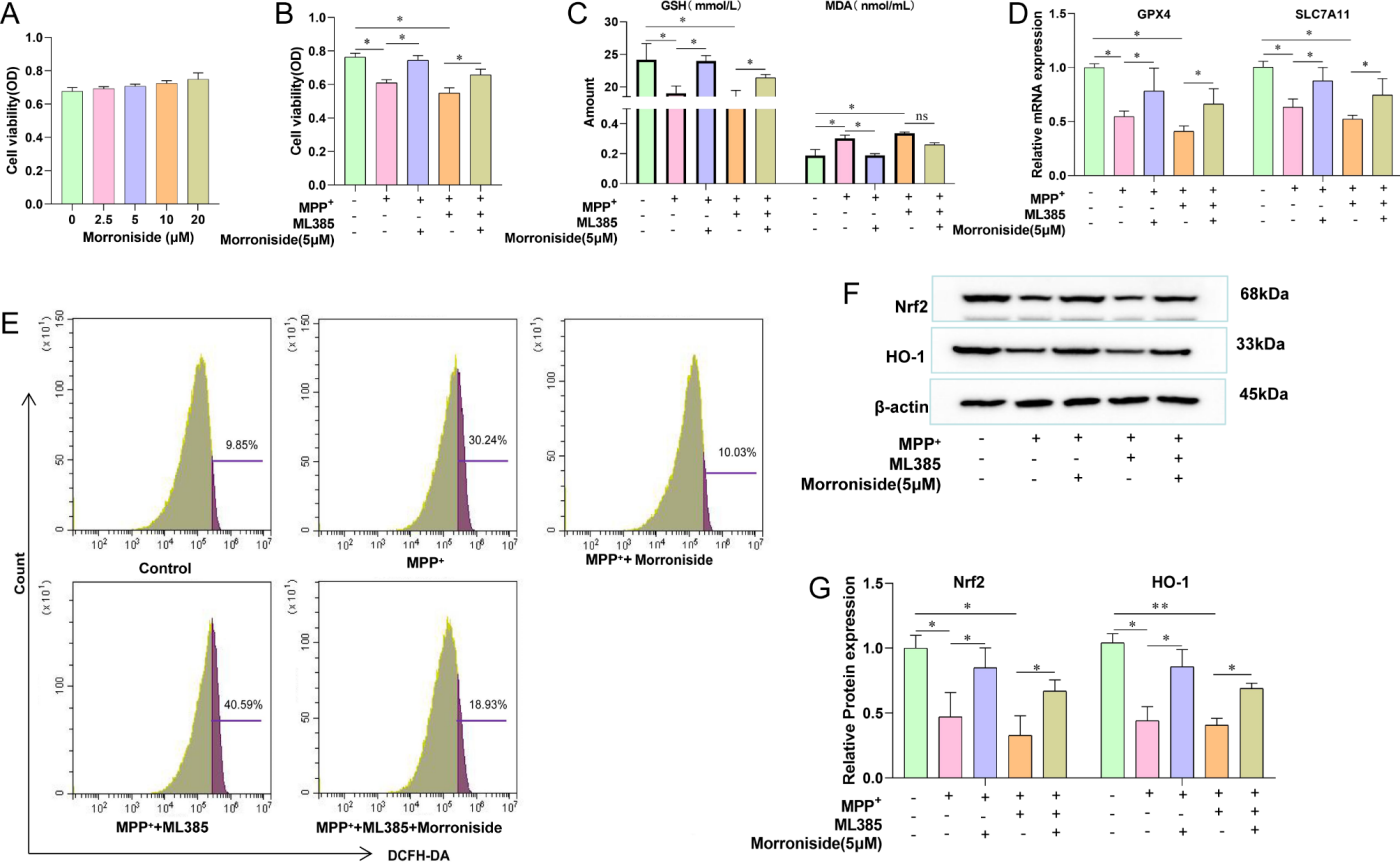
Effect of morroniside on oxidative stress in PC12 cells. **(A)** Effect of morroniside on the viability of PC12 cells by CCK-8. **(B)** Effect of morroniside or ML385 on the viability of PC12 cells by CCK-8. **(C)** GSH and MDA in PC12 cells through Elisa. **(D)** mRNA levels of GPX4 and SLC7A11. **(E)** Effect of morroniside or ML385 on ROS in PC12 cells using Flow Cytometry. **(F)** Levels of Nrf2 and HO-1 in PC12 cells by Western blot. **(G)** Relative expression of Nrf2 and HO-1. Data are shown as mean values ± SD, n = 3 per group, *p < 0.05, ns indicates *p* ≥ 0.05

PC12 cells have the general characteristics of neuroendocrine cells and have been widely used in in vitro studies on PD [29–31]. Morroniside at concentrations of 0 to 20 µM was not cytotoxic to PC12 cells (Fig. 4A). MPP^+^ could induce PC12 cell death, and when combined with the Nrf2 inhibitor ML385, it caused increased death of the PC12 cells. Morroniside at 5 µM could significantly reverse the cell death caused by MPP^+^ and ML385 (Fig. 4B). Moreover, the antioxidant capacity of PC12 cells was decreased, GSH was decreased, MDA was increased upon treatment with MPP^+^. Morroniside reverses the action of MPP+. MPP^+^ combined with ML385 resulted in a greater decrease in GSH and a greater increase in MDA levels, while 5 µM morroniside significantly reversed the adverse effects caused by MPP^+^ and ML385 down-regulated the mRNA levels of GPX4 and SLC7A11, while Morroniside could up-regulate the mRNA levels of GPX4 and SLC7A11 (Fig. 4D). ROS is a key factor of oxidative stress. ROS was significantly increased in PC12 cells treated with MPP^+^ compared to control cells. The combination of MPP^+^ with ML385 increased the levels of ROS in PC12 cells, while treatment with 5 µM morroniside could significantly reduce the levels of ROS (Fig. 4E). The levels of Nrf2 and HO-1 were reduced by MPP^+^, while a combination of MPP^+^ and ML385 brought about a greater decrease in these levels. Morroniside at a concentration of 5 µM could significantly reverse this decrease in the levels of Nrf2 and HO-1 (Fig. 4F and G). These results suggest that morroniside may regulate oxidative stress in PC12 cells through the Nrf2-ARE signaling pathway to affect ROS. ROS and lipid peroxidation of intracellular iron overload cause ferroptosis.

### Effect of morroniside on ferroptosis in PC12 cells

**Fig. 5:**
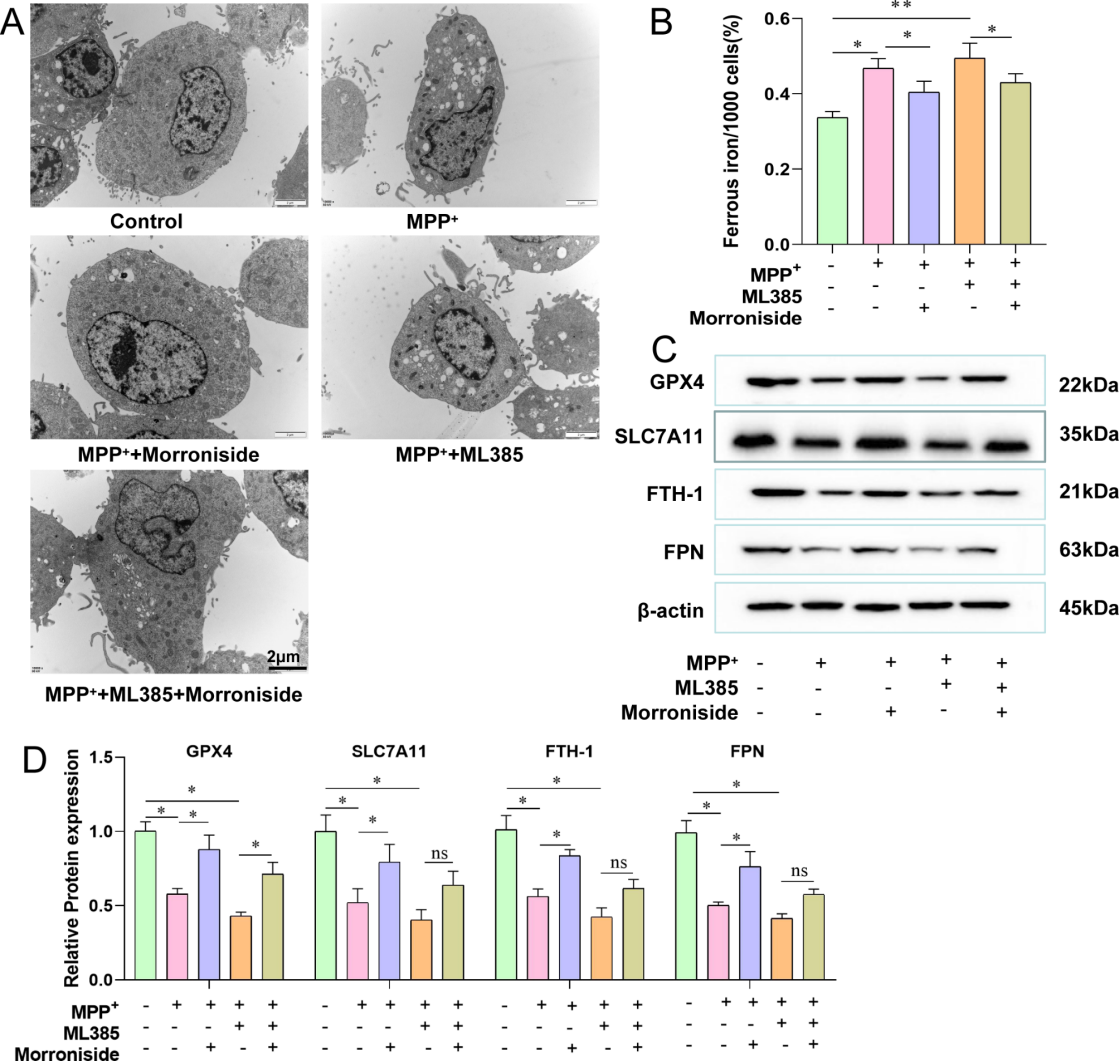
Morroniside inhibits ferroptosis in PC12 cells caused by MPP^+^. **(A)** Ultrastructure of PC12 cells under TEM. **(B)** Ferrous iron in PC12 cells. **(C)** Expression of GPX4, SLC7A11, FTH-1, and FPN in PC12 cells by Western blot. **(D)** Relative expression of GPX4, SLC7A11, FTH-1, and FPN. Data are shown as mean values ± SD, n = 3 per group, *p < 0.05, **p < 0.01, ns indicates *p* ≥ 0.05

PD is a degenerative disease of dopaminergic neurons, wherein iron deposition in the substantia nigra is an important factor. Compared with the control group, the MPP^+^ group or MPP^+^-ML385 combined group had mitochondria in the cells that were solidly constricted, the gaps between the cristae were widened, the mitochondria were darkened, some of the rough endoplasmic reticula were mildly dilated, and more vacuoles were visible. The ultrastructure of PC12 cells damaged by MPP^+^ combined with ML385 could be repaired with 5 µM morroniside (Fig. 5A). The ferrous iron content was highest in the MPP^+^ or MPP^+^-ML385 combined groups. In PC12 cells, treatment with 5 µM morroniside reduces the ferrous iron deposition induced by MPP^+^ and ML385 (Fig. 5B). MPP^+^ downregulated GPX4, SLC7A11, FTH-1, and FPN, which were further downregulated by the combination of MPP^+^ with ML385 (Fig. 5C and D). In contrast, morroniside promoted the expression of GPX4, SLC7A11, FTH-1, and FPN. Moreover, morroniside alone had no significant effect on GPX4, SLC7A11, FTH-1, or FPN. These results suggested that morroniside may regulate ferroptosis caused by MPP^+^ in PC12 cells through the upregulation of GPX4, SLC7A11, FTH-1, and FPN.

## Discussion

**Fig. 6:**
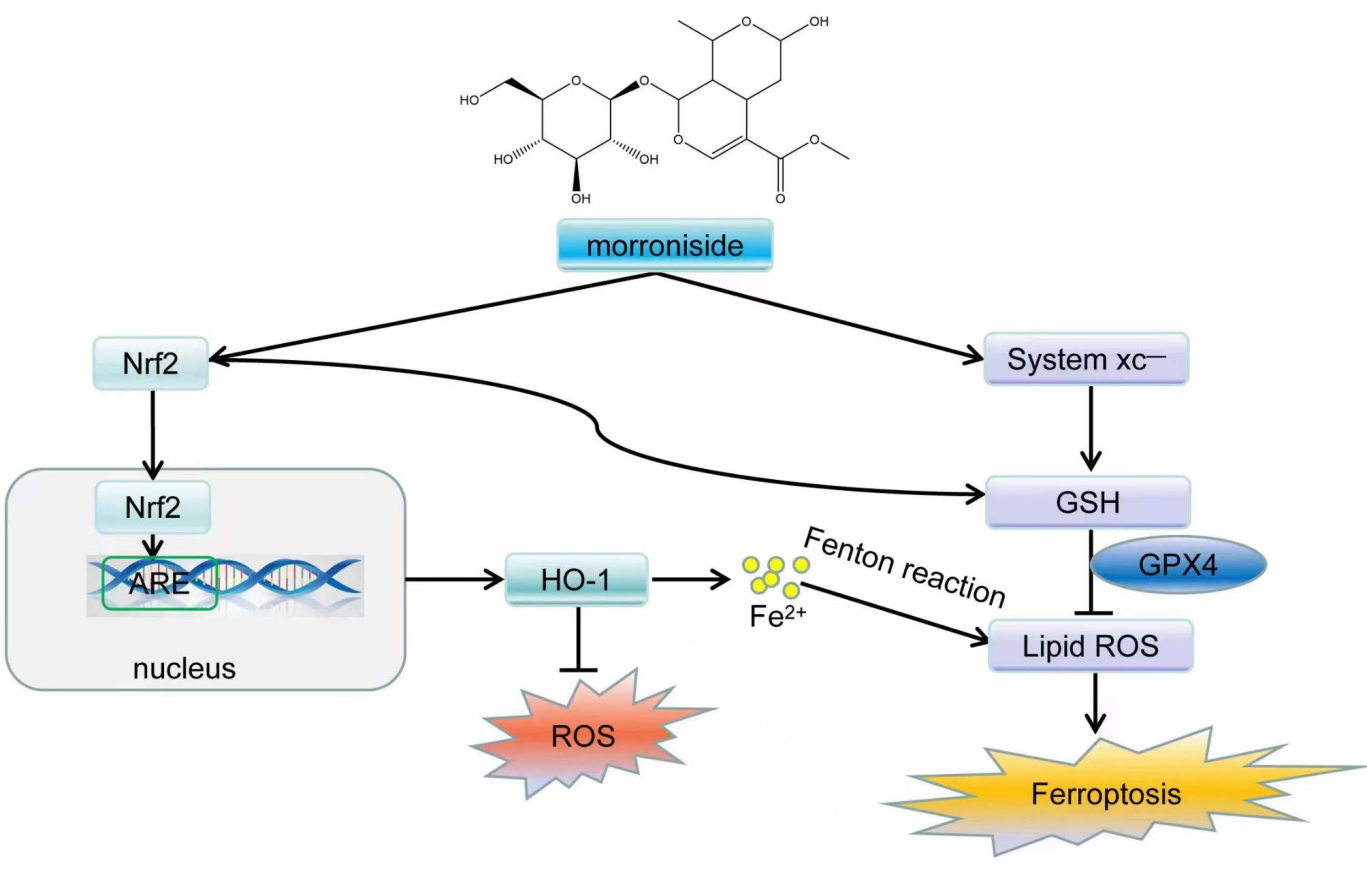
Schematic diagram of morroniside on neuroprotection

PD is a relatively common neurodegenerative disease in the elderly population, accompanied by a series of clinical syndromes with movement disorders as the main manifestation [1, 32]. Results of recent research indicate that several chronic diseases are associated with the accumulation of excessive free radicals in the body, and the relationship between PD, free radicals, and oxidative stress is increasingly becoming a hot topic of research [33, 34]. The cellular antioxidant capacity is reduced, and lipid-ROS accumulation leads to iron deposition in the nigrostriatal area, causing ferroptosis [35]. The pathogenesis of PD involves neuroinflammation, mitochondrial dysfunction and oxidative stress, of which mitochondrial dysfunction is widely recognised as being closely related to reactive oxygen species production and increased energy [36, 37]. Similar to ursolic acid and chlorogenic acid, morroniside also exhibited the neuroprotective effect in the MPTP-induced Parkinsonian mouse model [38, 39]. In this paper, we investigated the effects of morroniside on the behavioral and authentication abilities of PD mice and explored the enzyme TH, the rate-limiting enzyme for dopamine synthesis in the mouse brain substantia nigra, as well as the oxidative stress pathway and ferroptosis in vivo. We examined the protective effect of morroniside on mitochondria in vivo and in vitro. The effects of morroniside on oxidative stress and ferroptosis were also investigated in vitro in PC12 cells. The combination of in vivo and in vitro experiments confirmed that morroniside might regulate cellular ferroptosis via lipid peroxidation dependent on ROS and intracellular iron overload and affect PD development via Nrf2-ARE (Fig. 6).

MPTP is a toxin that potentially and selectively damages nigrostriatal dopamine neurons and can trigger PD in rodents [40]. After the administration of MPTP, mice showed abnormalities in locomotor ability and muscle tone, similar to the clinical manifestations of human PD [41]. Studies have demonstrated that PD mice prefer the open-field and slow-climbing time [42, 43]. The detection of behavioral deficits in PD mice using behavioral methods such as open-field and pole-climbing can aid in the potential therapeutic evaluation of drugs for PD. The pole-climbing time of morroniside-treated PD mice was significantly reduced, while the total distance and mean speed of open-field activity were significantly increased, indicating the occurrence of dyskinesia remission in PD mice.

The substantia nigra is the largest nucleus of the midbrain [44]. It is located on the dorsal side of the brain and runs through the entire length of the midbrain. It extends from the upper boundary of the pons gray matter to the hypothalamic basal nucleus plane. The absence of dopaminergic neurons in the substantia nigra is an important pathophysiological basis of PD [45]. Moreover, TH is a key rate-limiting enzyme in the synthesis of catecholamine neurotransmitters (dopamine) [46]. The changes in the content of TH in the brain directly affect the biosynthesis of levodopa in the substantial nigra-striatum system [47]. The decrease in the level or activity of dopaminergic neurons can decrease the synthesis and secretion of dopamine neurotransmitters, which contributes to the pathogenesis of PD [48]. As a result, changes in TH content can reflect the onset and progression of PD. This study found that intraperitoneal injection of MPTP could significantly reduce TH in the substantia nigra of mice, resulting in Parkinson’s-like symptoms. Morroniside can reverse the decrease in neuron number caused by MPTP. It may also significantly increase the expression of TH in the brain of the PD mouse model, increasing the content of dopamine neurotransmitters in the brain and improving symptoms of motor disorders in the PD rat model. Moreover, previous studies have reported that MPTP causes a decrease in the TH levels in the brain by inducing ROS and inflammatory responses, which, being a major parameter of oxidative stress, can affect the activity and function of dopaminergic neurons [49].

The transcription factor Nrf2 is a key factor in the cellular oxidative stress response and is transferred from the cytoplasm into the nucleus under oxidative stress. It then binds to the ARE to regulate the expression of antioxidant proteins, thereby enhancing cellular resistance to oxidative stress [11]. Siebert [50] reported that Nrf2 was activated by tert-butyl hydroquinone (tBHQ) and protected against 6-hydroxy dopamine (6-OH-DA)-induced damage to nigrostriatal coculture tissues. In PD, a slight increase in the HO-1 protein and increased immune reactivity in the Lewy body were observed in dopamine neurons in the substantia nigra compacta [51]. Increased expression of HO-1 counteracts oxidative stress and neuroinflammatory damage in both in vitro and in vivo model experiments on PD [52]. In this paper, MPTP inhibited Nrf2 and HO-1 in the brain substantia nigra and the PC12 cells. Morroniside, on the other hand, induced the expression of Nrf2 and HO-1 both in vivo and in vitro, implying that morroniside could regulate the levels of oxidative stress factors via Nrf2/HO-1. The most important oxidative stress parameters are GSH and MDA. Morroniside modulated both GSH and MDA in both in vivo and in vitro experiments, restoring the parameters to the levels observed in the control group. Furthermore, MPP^+^-induced MDA and ROS in PC12 cells were exacerbated by the Nrf2-inhibitor ML385, which was reversed by morroniside treatment, demonstrating the importance of Nrf2 in morroniside-mediated PD mitigation. ROS generated during dopamine synthesis, low levels of GSH, and increased concentration of iron ions are the main factors leading to the death of the brain neurons in PD patients [53]. The lack or decrease in levels of GSH will increase the susceptibility to oxidative stress, which may lead to the occurrence of diseases such as PD, AD, and tumors [54]. Free radicals can react with unsaturated fatty acids on the cell membrane, leading to lipid peroxidation; thus, producing abundant MDA [55]. Previous studies have shown that the function of mitochondria is critical for ferroptosis [56]. Mitochondria are important organelles within the cell and their main function is to provide a large amount of energy through oxidative phosphorylation of the respiratory chain. In addition, mitochondria could also act as hubs for a number of metabolic or signalling pathways, particularly fatty acid metabolism [57]. Importantly, morroniside could repair mitochondrial damage both in vitro and in vivo, suggesting that it may restore the mitochondrial respiratory chain and mitochondrial function to reduce ROS production. Free dopamine in the cytoplasm produces ROS and the toxic dopamine quinone (DAQ) via autoxidation [58]. DAQ affects mitochondrial morphology and induces the depolarization of the mitochondrial membrane, which induces the opening of the mitochondrial membrane permeability transition pore (mPTP) and increases the sensitivity of dopaminergic neurons to injury [59].

Recently, several studies have pointed out that oxidative stress [60, 61], abnormal glutamate metabolism [62], and lipid peroxide accumulation in PD models are quite similar to ferroptosis. Erastin can cause ferroptosis by inhibiting the system Xc- (SLC7A11: SLC3A2) activity, reducing the intracellular Cys content, blocking the synthesis of GSH, and accumulating intracellular lipid ROS [63]. Therefore, ferroptosis may play an important role in the development of PD. GPX4 is a GSH-promoted reduction reaction of peroxidized lipids, preventing ferroptosis [64]. FTH1 is an iron storage protein complex, and its reduced levels along with an excess free iron can cause disorders of lipid metabolism [65]. The membrane iron transporter protein FPN can maintain cellular iron stability across iron metabolic pathway [66, 67]. Morroniside reduced iron content in substantia nigra and inhibited ferroptosis in PD mice in the current study. Morroniside reduced iron content in PC12 cells and inhibited ferroptosis caused by MPP^+^, which was consistent with in vivo experiments. Morroniside also restored the proteins GPX4, SLC7A11, FTH-1, and FPN, which became abnormal during ferroptosis in vivo and in vitro experiments. Morroniside can inhibit the development and progression of PD by inhibiting iron aggregation. However, the relationship between Nrf2 and ferroptosis has not been as intensively studied, and further research will be conducted at a later stage to confirm this inference.

## Conclusion

In conclusion, this study has demonstrated that morroniside improves the behavioral profile of mice with MPTP-induced dopaminergic neuronal damage and mitochondrial damage, induces the expression of TH, and regulates the level of oxidative stress in the body via the Nrf2-ARE pathway. It also restores oxidative homeostasis in the brains of PD mouse models, protects against mitochondrial damage, inhibits the production of the lipid metabolite MDA, increases the content of the free radical-scavenger GSH, decreases iron content, and inhibits ferroptosis. Furthermore, the effect of morroniside on ferroptosis was confirmed in PC12 cells. The Nrf2-ARE pathway, which is linked to the pathogenesis of PD, promotes antioxidant levels and induces the expression of iron-regulated proteins to protect dopaminergic neurons in the substantia nigra, delaying the progression of the disease. Ferroptosis is a unique form of programmed cell death caused by lipid peroxidation dependent on ROS and intracellular iron overload, but many of its physiological roles remain to be clarified.

## Electronic supplementary material

Below is the link to the electronic supplementary material.

**Additional file 1**

**Additional file 2**

## Data Availability

The raw data supporting the conclusions of this article will be made available by the authors without undue reservation.

## References

[1] (2021) Parkinson disease-associated cognitive impairment. Nat Rev Dis Primers 7:46. 10.1038/s41572-021-00286-x. 10.1038/s41572-021-00280-3 34210995

[2] Rai SN, Singh P, Varshney R, Chaturvedi VK, Vamanu E, Singh MP, Singh BK (2021). Promising drug targets and associated therapeutic interventions in Parkinson’s disease. Neural Regen Res.

[3] Rai SN, Chaturvedi VK, Singh P, Singh BK, Singh MP (2020). Mucuna pruriens in Parkinson’s and in some other diseases: recent advancement and future prospective. 3 Biotech.

[4] Rai SN, Singh P (2020). Advancement in the modelling and therapeutics of Parkinson’s disease. J Chem Neuroanat.

[5] Schweitzer JS, Song B, Kim KS (2021). A step closer to autologous cell therapy for Parkinson’s disease. Cell Stem Cell.

[6] Vijiaratnam N, Simuni T, Bandmann O, Morris HR, Foltynie T (2021). Progress towards therapies for disease modification in Parkinson’s disease. Lancet Neurol.

[7] Ghosh A, Tyson T, George S, Hildebrandt EN, Steiner JA, Madaj Z, Schulz E, Machiela E, McDonald WG, Escobar Galvis ML, Kordower JH, Van Raamsdonk JM, Colca JR, Brundin P (2016). Mitochondrial pyruvate carrier regulates autophagy, inflammation, and neurodegeneration in experimental models of Parkinson’s disease. Sci Transl Med.

[8] de Bie R, Clarke CE, Espay AJ, Fox SH, Lang AE (2020). Initiation of pharmacological therapy in Parkinson’s disease: when, why, and how. Lancet Neurol.

[9] Di Gioia S, Trapani A, Cassano R, Di Gioia ML, Trombino S, Cellamare S, Bolognino I, Hossain MN, Sanna E, Trapani G, Conese M (2021). Nose-to-brain delivery: a comparative study between carboxymethyl chitosan based conjugates of dopamine. Int J Pharm.

[10] Dionísio PA, Amaral JD, Rodrigues C (2021). Oxidative stress and regulated cell death in Parkinson’s disease. Ageing Res Rev.

[11] Zhang W, Feng C, Jiang H (2021). Novel target for treating Alzheimer’s Diseases: crosstalk between the Nrf2 pathway and autophagy. Ageing Res Rev.

[12] Jazwa A, Rojo AI, Innamorato NG, Hesse M, Fernández-Ruiz J, Cuadrado A (2011). Pharmacological targeting of the transcription factor Nrf2 at the basal ganglia provides disease modifying therapy for experimental parkinsonism. Antioxid Redox Signal.

[13] Kaidery NA, Banerjee R, Yang L, Smirnova NA, Hushpulian DM, Liby KT, Williams CR, Yamamoto M, Kensler TW, Ratan RR, Sporn MB, Beal MF, Gazaryan IG, Thomas B (2013). Targeting Nrf2-mediated gene transcription by extremely potent synthetic triterpenoids attenuate dopaminergic neurotoxicity in the MPTP mouse model of Parkinson’s disease. Antioxid Redox Signal.

[14] Clements CM, McNally RS, Conti BJ, Mak TW, Ting JP (2006). DJ-1, a cancer- and Parkinson’s disease-associated protein, stabilizes the antioxidant transcriptional master regulator Nrf2. Proc Natl Acad Sci U S A.

[15] Dodson M, Castro-Portuguez R, Zhang DD (2019). NRF2 plays a critical role in mitigating lipid peroxidation and ferroptosis. Redox Biol.

[16] Do Van B, Gouel F, Jonneaux A, Timmerman K, Gelé P, Pétrault M, Bastide M, Laloux C, Moreau C, Bordet R, Devos D, Devedjian JC (2016). Ferroptosis, a newly characterized form of cell death in Parkinson’s disease that is regulated by PKC. Neurobiol Dis.

[17] Angelova PR, Esteras N, Abramov AY (2021). Mitochondria and lipid peroxidation in the mechanism of neurodegeneration: finding ways for prevention. Med Res Rev.

[18] Wu H, Wang F, Ta N, Zhang T, Gao W. The multifaceted regulation of Mitochondria in Ferroptosis. Life (Basel). 2021;11. 10.3390/life11030222. 10.3390/life11030222 PMC8001967 33801920

[19] Tang D, Kang R, Berghe TV, Vandenabeele P, Kroemer G (2019). The molecular machinery of regulated cell death. Cell Res.

[20] Islam MT (2017). Oxidative stress and mitochondrial dysfunction-linked neurodegenerative disorders. Neurol Res.

[21] Ye XS, He J, Xu JK, He XL, Xia CY, Yan Y, Lian WW, Zhang WK (2020). Undescribed morroniside-like secoiridoid diglycosides with α-glucosidase inhibitory activity from Corni Fructus. Phytochemistry.

[22] Zeng G, Ding W, Li Y, Sun M, Deng L (2018). Morroniside protects against cerebral ischemia/reperfusion injury by inhibiting neuron apoptosis and MMP2/9 expression. Exp Ther Med.

[23] Duan FX, Shi YJ, Chen J, Song X, Shen L, Qi Q, Ding SQ, Wang QY, Wang R, Lü HZ, Hu JG (2021). The neuroprotective role of morroniside against spinal cord injury in female rats. Neurochem Int.

[24] Park C, Cha HJ, Lee H, Kim GY, Choi YH (2021). The regulation of the TLR4/NF-κB and Nrf2/HO-1 signaling pathways is involved in the inhibition of lipopolysaccharide-induced inflammation and oxidative reactions by morroniside in RAW 264.7 macrophages. Arch Biochem Biophys.

[25] Wang W, Xu J, Li L, Wang P, Ji X, Ai H, Zhang L, Li L (2010). Neuroprotective effect of morroniside on focal cerebral ischemia in rats. Brain Res Bull.

[26] Rodrigues AL, Rocha JB, Mello CF, Souza DO (1996). Effect of perinatal lead exposure on rat behaviour in open-field and two-way avoidance tasks. Pharmacol Toxicol.

[27] Matsuura K, Kabuto H, Makino H, Ogawa N (1997). Pole test is a useful method for evaluating the mouse movement disorder caused by striatal dopamine depletion. J Neurosci Methods.

[28] Tai S, Zheng Q, Zhai S, Cai T, Xu L, Yang L, Jiao L, Zhang C (2020). Alpha-lipoic acid mediates clearance of Iron Accumulation by Regulating Iron Metabolism in a Parkinson’s Disease Model Induced by 6-OHDA. Front Neurosci.

[29] Lee J, Song K, Huh E, Oh MS, Kim YS (2018). Neuroprotection against 6-OHDA toxicity in PC12 cells and mice through the Nrf2 pathway by a sesquiterpenoid from Tussilago farfara. Redox Biol.

[30] Cui W, Zhan Y, Shao X, Fu W, Xiao D, Zhu J, Qin X, Zhang T, Zhang M, Zhou Y, Lin Y (2019) Neuroprotective and Neurotherapeutic Effects of Tetrahedral Framework Nucleic Acids on Parkinson’s Disease in Vitro. ACS Appl Mater Interfaces 11:32787–32797. 10.1021/acsami.9b10308. 10.1021/acsami.9b10308 31424187

[31] Liu J, Liu C, Zhang J, Zhang Y, Liu K, Song JX, Sreenivasmurthy SG, Wang Z, Shi Y, Chu C, Zhang Y, Wu C, Deng X, Liu X, Song J, Zhuang R, Huang S, Zhang P, Li M, Wen L, Zhang YW, Liu G (2020). A self-assembled α-Synuclein nanoscavenger for Parkinson’s Disease. ACS Nano.

[32] Tolosa E, Garrido A, Scholz SW, Poewe W (2021). Challenges in the diagnosis of Parkinson’s disease. Lancet Neurol.

[33] Trist BG, Hare DJ, Double KL (2019). Oxidative stress in the aging substantia nigra and the etiology of Parkinson’s disease. Aging Cell.

[34] Robea MA, Balmus IM, Ciobica A, Strungaru S, Plavan G, Gorgan LD, Savuca A, Nicoara M (2020). Parkinson’s Disease-Induced zebrafish models: focussing on oxidative stress implications and sleep processes. Oxid Med Cell Longev.

[35] Guiney SJ, Adlard PA, Bush AI, Finkelstein DI, Ayton S (2017). Ferroptosis and cell death mechanisms in Parkinson’s disease. Neurochem Int.

[36] Ren ZL, Wang CD, Wang T, Ding H, Zhou M, Yang N, Liu YY, Chan P (2019). Ganoderma lucidum extract ameliorates MPTP-induced parkinsonism and protects dopaminergic neurons from oxidative stress via regulating mitochondrial function, autophagy, and apoptosis. Acta Pharmacol Sin.

[37] Wei Z, Li X, Li X, Liu Q, Cheng Y (2018). Oxidative stress in Parkinson’s Disease: a systematic review and Meta-analysis. Front Mol Neurosci.

[38] Rai SN, Zahra W, Singh SS, Birla H, Keswani C, Dilnashin H, Rathore AS, Singh R, Singh RK, Singh SP (2019). Anti-inflammatory activity of Ursolic Acid in MPTP-Induced Parkinsonian Mouse Model. Neurotox Res.

[39] Singh SS, Rai SN, Birla H, Zahra W, Rathore AS, Dilnashin H, Singh R, Singh SP (2020). Neuroprotective effect of Chlorogenic Acid on mitochondrial dysfunction-mediated apoptotic death of DA neurons in a Parkinsonian Mouse Model. Oxid Med Cell Longev.

[40] Prediger RD, Aguiar AS, Moreira EL, Matheus FC, Castro AA, Walz R, De Bem AF, Latini A, Tasca CI, Farina M, Raisman-Vozari R (2011). The intranasal administration of 1-methyl-4-phenyl-1,2,3,6-tetrahydropyridine (MPTP): a new rodent model to test palliative and neuroprotective agents for Parkinson’s disease. Curr Pharm Des.

[41] Tillerson JL, Caudle WM, Reverón ME, Miller GW (2002). Detection of behavioral impairments correlated to neurochemical deficits in mice treated with moderate doses of 1-methyl-4-phenyl-1,2,3,6-tetrahydropyridine. Exp Neurol.

[42] Liu SM, Li XZ, Zhang SN, Yang ZM, Wang KX, Lu F, Wang CZ, Yuan CS (2018). Acanthopanax senticosus protects structure and function of Mesencephalic Mitochondria in A Mouse Model of Parkinson’s Disease. Chin J Integr Med.

[43] Han QQ, Fu Y, Le JM, Pilot A, Cheng S, Chen PQ, Wu H, Wan GQ, Gu XF (2021). Electroacupuncture may alleviate behavioral defects via modulation of gut microbiota in a mouse model of Parkinson’s disease. Acupunct Med.

[44] Misgeld U (2004). Innervation of the substantia nigra. Cell Tissue Res.

[45] Michel PP, Hirsch EC, Hunot S (2016). Understanding dopaminergic cell death pathways in Parkinson Disease. Neuron.

[46] Daubner SC, Le T, Wang S (2011). Tyrosine hydroxylase and regulation of dopamine synthesis. Arch Biochem Biophys.

[47] Salvatore MF, McInnis TR, Cantu MA, Apple DM, Pruett BS (2019). Tyrosine hydroxylase inhibition in Substantia Nigra decreases Movement frequency. Mol Neurobiol.

[48] Nagatsu T, Nagatsu I (2016). Tyrosine hydroxylase (TH), its cofactor tetrahydrobiopterin (BH4), other catecholamine-related enzymes, and their human genes in relation to the drug and gene therapies of Parkinson’s disease (PD): historical overview and future prospects. J Neural Transm (Vienna).

[49] Rekaik H, Blaudin de Thé FX, Prochiantz A, Fuchs J, Joshi RL (2015). Dissecting the role of Engrailed in adult dopaminergic neurons–insights into Parkinson disease pathogenesis. FEBS Lett.

[50] Siebert A, Desai V, Chandrasekaran K, Fiskum G, Jafri MS (2009). Nrf2 activators provide neuroprotection against 6-hydroxydopamine toxicity in rat organotypic nigrostriatal cocultures. J Neurosci Res.

[51] Schipper HM, Liberman A, Stopa EG (1998). Neural heme oxygenase-1 expression in idiopathic Parkinson’s disease. Exp Neurol.

[52] Chen PC, Vargas MR, Pani AK, Smeyne RJ, Johnson DA, Kan YW, Johnson JA (2009). Nrf2-mediated neuroprotection in the MPTP mouse model of Parkinson’s disease: critical role for the astrocyte. Proc Natl Acad Sci U S A.

[53] Chinta SJ, Andersen JK (2008). Redox imbalance in Parkinson’s disease. Biochim Biophys Acta.

[54] Ballatori N, Krance SM, Notenboom S, Shi S, Tieu K, Hammond CL (2009). Glutathione dysregulation and the etiology and progression of human diseases. Biol Chem.

[55] Tang D, Chen X, Kang R, Kroemer G (2021). Ferroptosis: molecular mechanisms and health implications. Cell Res.

[56] Gao M, Yi J, Zhu J, Minikes AM, Monian P, Thompson CB, Jiang X (2019). Role of Mitochondria in Ferroptosis. Mol Cell.

[57] Sedlackova L, Korolchuk VI (2019). Mitochondrial quality control as a key determinant of cell survival. Biochim Biophys Acta Mol Cell Res.

[58] Miyazaki I, Asanuma M (2008). Dopaminergic neuron-specific oxidative stress caused by dopamine itself. Acta Med Okayama.

[59] Biosa A, Arduini I, Soriano ME, Giorgio V, Bernardi P, Bisaglia M, Bubacco L (2018). Dopamine Oxidation Products as mitochondrial endotoxins, a potential molecular mechanism for preferential neurodegeneration in Parkinson’s Disease. ACS Chem Neurosci.

[60] Gao HM, Zhou H, Hong JS (2012). NADPH oxidases: novel therapeutic targets for neurodegenerative diseases. Trends Pharmacol Sci.

[61] Brenner S (2014). Parkinson’s disease may be due to failure of melanin in the Substantia Nigra to produce molecular hydrogen from dissociation of water, to protect the brain from oxidative stress. Med Hypotheses.

[62] Niswender CM, Conn PJ (2010). Metabotropic glutamate receptors: physiology, pharmacology, and disease. Annu Rev Pharmacol Toxicol.

[63] Dixon SJ, Lemberg KM, Lamprecht MR, Skouta R, Zaitsev EM, Gleason CE, Patel DN, Bauer AJ, Cantley AM, Yang WS, Morrison B, Stockwell BR (2012). Ferroptosis: an iron-dependent form of nonapoptotic cell death. Cell.

[64] Ursini F, Maiorino M (2020). Lipid peroxidation and ferroptosis: the role of GSH and GPx4. Free Radic Biol Med.

[65] Tian Y, Lu J, Hao X, Li H, Zhang G, Liu X, Li X, Zhao C, Kuang W, Chen D, Zhu M (2020). FTH1 inhibits ferroptosis through Ferritinophagy in the 6-OHDA model of Parkinson’s Disease. Neurotherapeutics.

[66] Geng N, Shi BJ, Li SL, Zhong ZY, Li YC, Xua WL, Zhou H, Cai JH (2018). Knockdown of ferroportin accelerates erastin-induced ferroptosis in neuroblastoma cells. Eur Rev Med Pharmacol Sci.

[67] Reichert CO, de Freitas FA, Sampaio-Silva J, Rokita-Rosa L, Barros PL, Levy D, Bydlowski SP. Ferroptosis mechanisms involved in neurodegenerative Diseases. Int J Mol Sci. 2020;21. 10.3390/ijms21228765. 10.3390/ijms21228765 PMC7699575 33233496

